# Supplementary material for: Broad fault zones enable deep fluid transport and limit earthquake magnitudes
Source: Nat Commun. 2023 Sep 16;14:5748. doi: 10.1038/s41467-023-41403-6 (PMC10505138; doi:10.1038/s41467-023-41403-6)
Supplement: Supplementary file 1 — Supplementary Information [file 41467_2023_41403_MOESM1_ESM.pdf]

Supplementary Table 1 | b-values for OBS catalogue for different magnitude cut-off values, as derived by maximum likelihood estimation (MLE[54]) and the repeated median approach (RM[55]), with their standard errors.

	Mc=2.0	Mc=2.1	Mc=2.2	Mc=2.3	Mc=2.4	Mc=2.5	Mc=2.6	Mc=2.7
MLE	**0.65**±0.03	**0.70**±0.03	**0.72**±0.04	**0.76**±0.04	**0.78**±0.04	**0.80**±0.05	**0.83**±0.05	**0.86**±0.06
RM	**0.83**±0.08	**0.82**±0.09	**0.82**±0.09	**0.83**±0.09	**0.84**±0.10	**0.83**±0.10	**0.83**±0.11	**0.82**±0.11

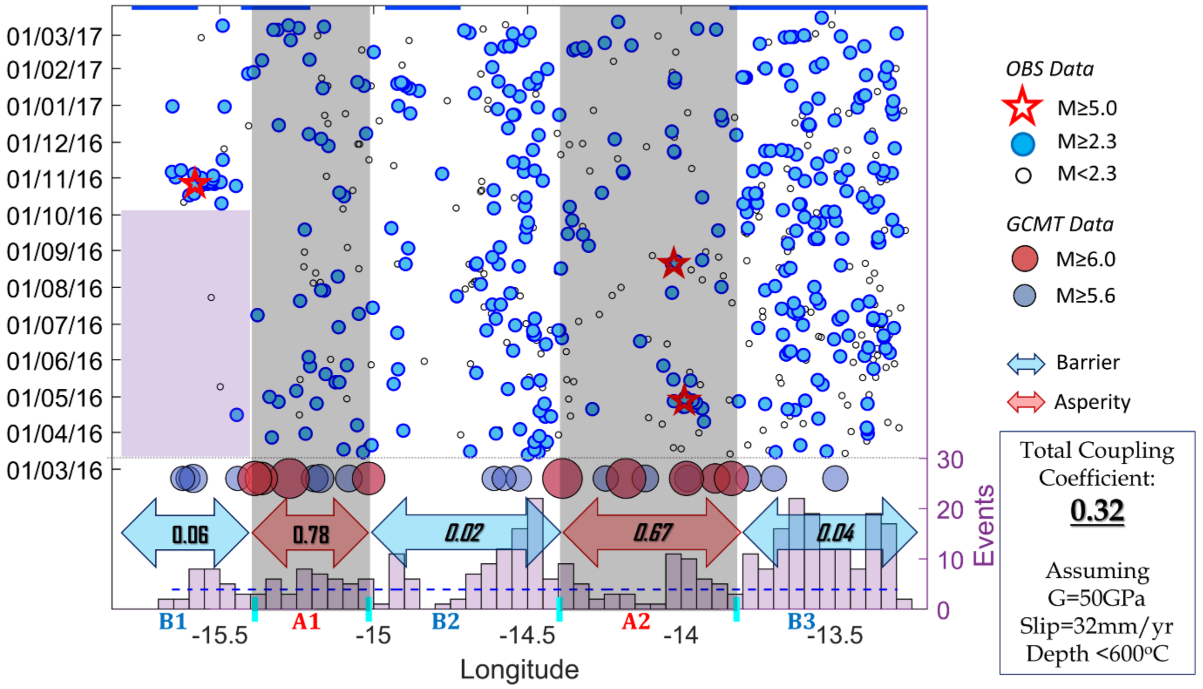

Supplementary Fig. 1 | Temporal and along strike microseismicity distribution. The blue circles indicate the OBS events above $M_C = 2.3$, whereas the white circles denote all located events. The largest OBS events with $5.0 \leq M_L \leq 5.4$ are depicted by red stars. The histogram shows the number of OBS events per 0.05° longitudinal bins. The purple box indicates a spatio-temporal microseismicity gap occurred at least 7 months before the M5.4 event sequence in barrier zone, B1. The large blue and red circles show the historical events with $M_W \geq 5.6$ and $M_W \geq 6.0$, respectively[31]. The grey shaded areas correspond to asperities (A1 and A2) which separate the barriers (B1, B2, B3). The number in the arrows show the seismically released moment fraction for these segments. The blue lines at upper horizontal margin indicate the location of the positive flower structures.

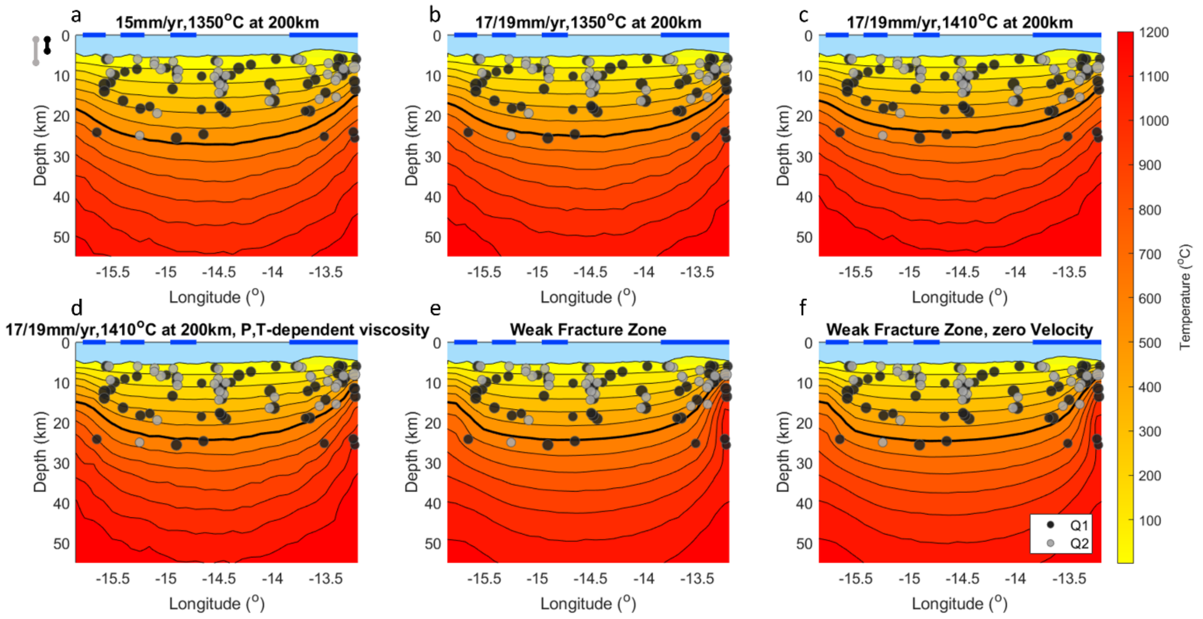

Supplementary Fig. 2 | Alternative thermal models. a) Spreading rate 15 mm/yr and 1350 °C at 200 km depth, b) unequal spreading rate 17 mm/yr (west-east) and 19 mm/yr (east-west) and 1350 °C at 200 km depth, c) unequal spreading rate 17 mm/yr (west-east) and 19 mm/yr (east-west) and 1410 °C at 200 km depth, d) unequal spreading rate 17 mm/yr (west-east) and 19 mm/yr (east-west), 1410 °C at 200 km depth and pressure-temperature dependent viscosity, e) Weak fracture zone model, f) Weak fracture zone model, zero velocity. The depth determination quality (Q1 and Q2) is indicated as dark and light circles, respectively. Mean vertical uncertainties are 3 km for Q1 events and 6 km for Q2 events (black and grey vertical error bar, respectively, shown at the upper left corner). The horizontal blue lines denote the location of the transpressional flower structures. The isolines have 100 °C difference with the bold one representing the 600 °C isotherm.

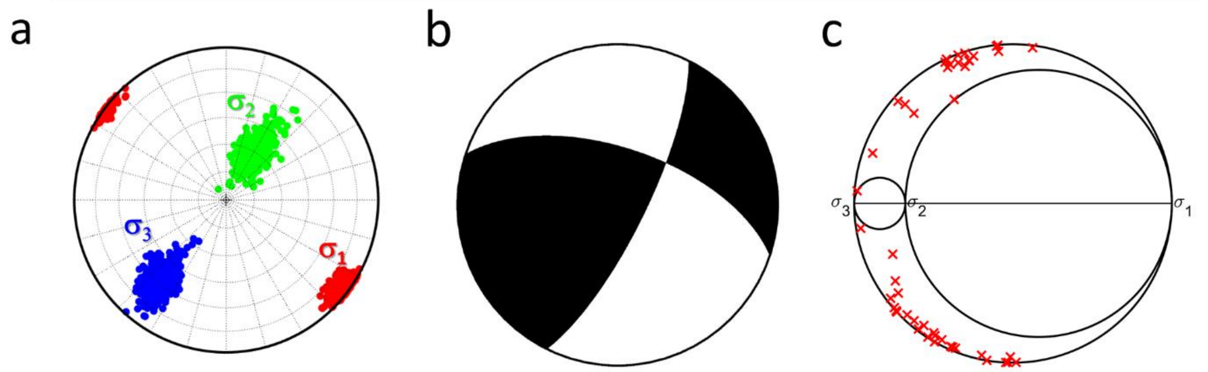

Supplementary Fig. 3 | Stress inversion results. (a) Confidence intervals of the principal stresses orientation, for the 47 best quality mechanisms. (b) Principal focal mechanism, shown as lower hemisphere projection, with compression denoted by black. (c) Mohr's circle plot showing the relative magnitudes of the principal stresses, with the positions of the faults indicated by red crosses.

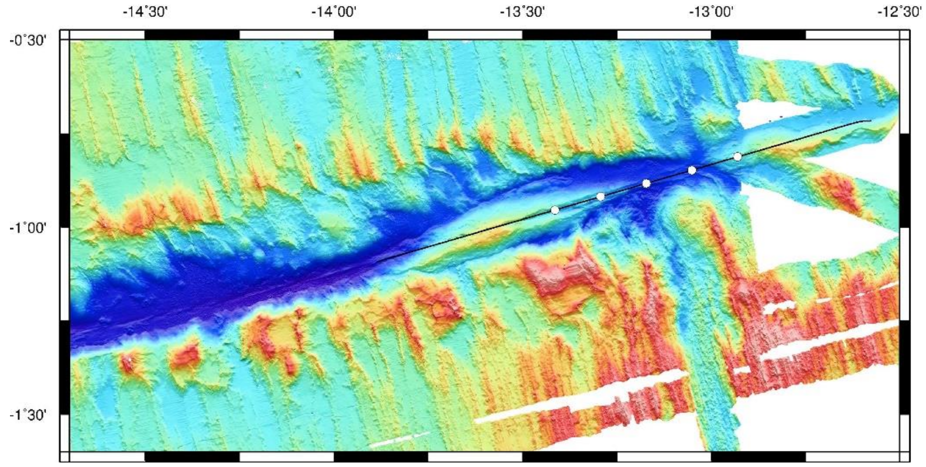

Supplementary Fig. 4 | Active source seismic profile. Location map of the active source seismic profile conducted at the eastern Ridge-Transform intersection of the Chain Transform Fault aboard the French N/O Pourquoi-Pas?.

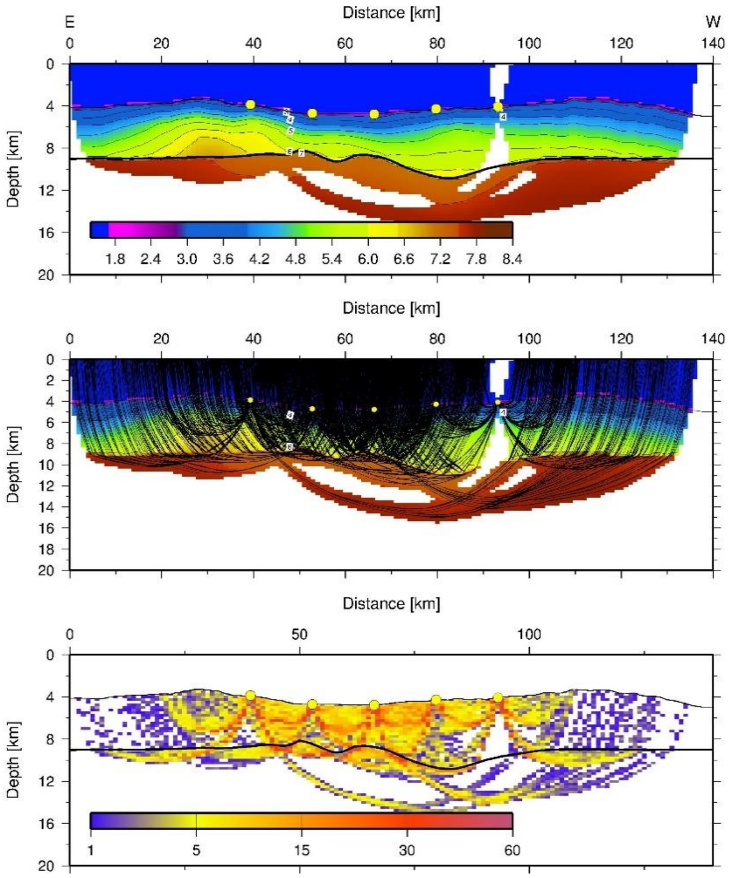

Supplementary Fig. 5 | Tomographic inversion. Results from the tomographic travel time inversion (top) seismic velocity model masked with available ray coverage; (middle) ray coverage; (bottom) cell hit counts (derivative weight sum) of the tomographic inversion.

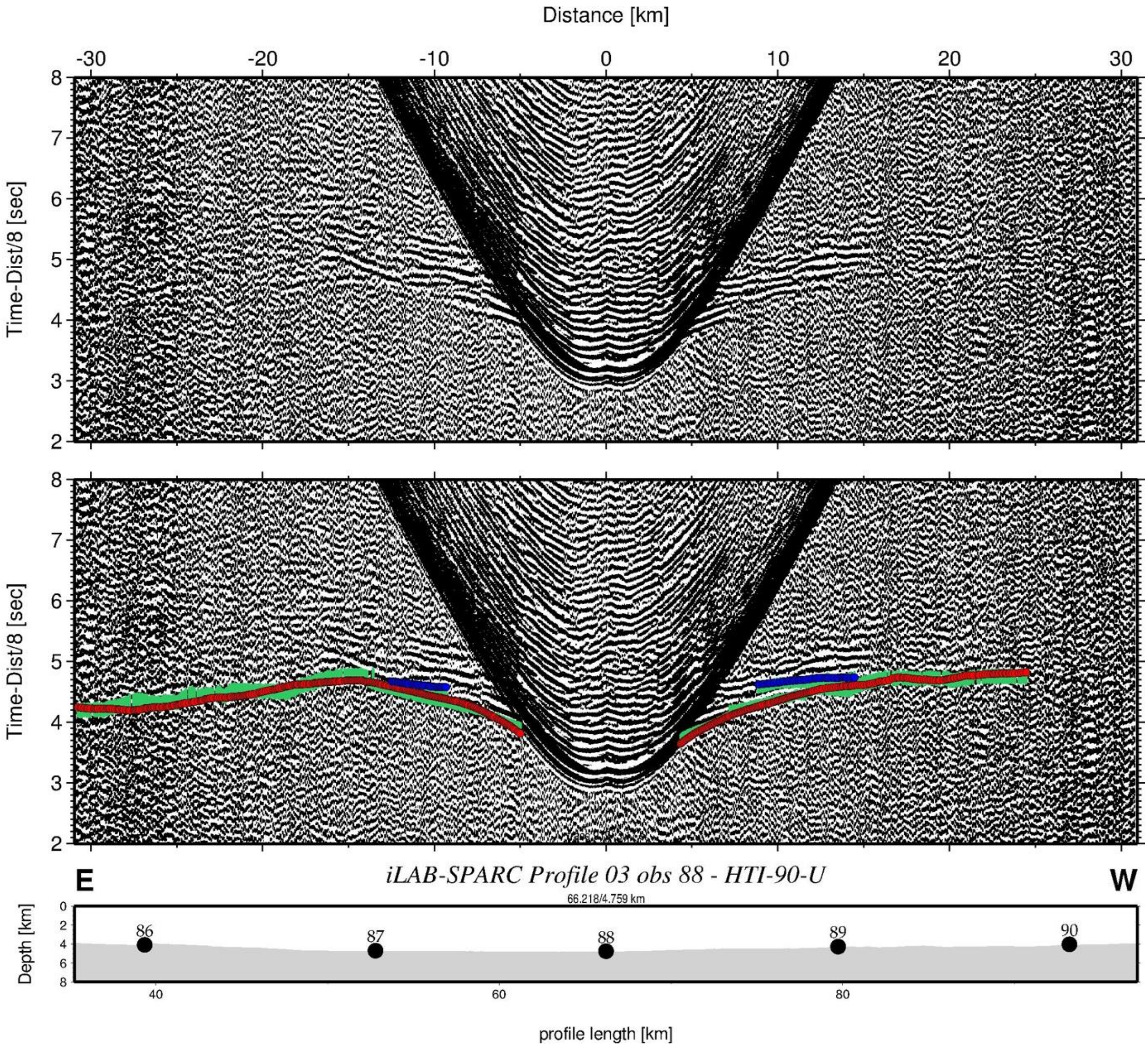

Supplementary Fig. 6 | Active source reflection data. Picks (green) and calculated arrival times are shown. The direct phase is shown in red and the wide-angle reflection is shown in blue. Only those reflections that are best defined are used.

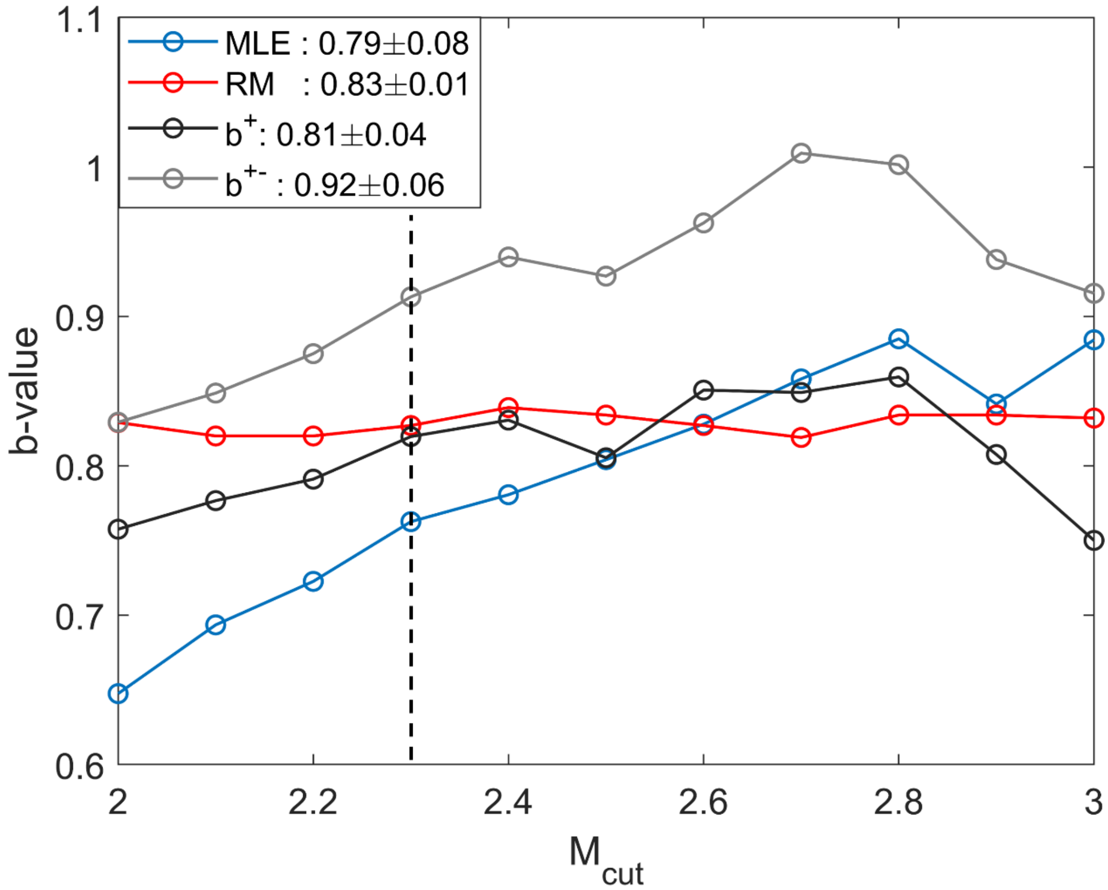

Supplementary Fig. 7 | b-values estimation. b-value of the OBS catalogue estimated by different techniques, including: MLE, maximum likelihood estimate[54]; RM, repeated medians technique[55]; b^+, b-positive estimate[56]; b^{+-}, b-positive/b-negative estimate[56]. The legend shows the mean values within the given range and their standard deviation. The vertical dashed line depicts the selected M_C = 2.3.
