# Supplementary material for: Broad fault zones enable deep fluid transport and limit earthquake magnitudes
Source: Nat Commun. 2023 Sep 16;14:5748. doi: 10.1038/s41467-023-41403-6 (PMC10505138; doi:10.1038/s41467-023-41403-6)
Supplement: Supplementary file 3 — Description of Additional Supplementary Files [file 41467_2023_41403_MOESM3_ESM.pdf]

Description of Additional Supplementary Files

File Name: Supplementary Data 1
Description: Active source velocity model.

File Name: Supplementary Movie 1
Description: Along-strike variation of b-value in Chain. Different moving windows are considered, ranging from N=50 to N=100 events, advancing by 1 event, superimposed over a bathymetry map of Chain. Each calculation is shown as a coloured circle, centred at the longitude of the central event in each window. The events shown as white circles and used for b-value calculations correspond to the complete dataset ($M_L \geq 2.3$) along the transform valley.
