# Supplementary figures and images for: Broad fault zones enable deep fluid transport and limit earthquake magnitudes
Source: Nat Commun. 2023 Sep 16;14:5748. doi: 10.1038/s41467-023-41403-6 (PMC10505138; doi:10.1038/s41467-023-41403-6)

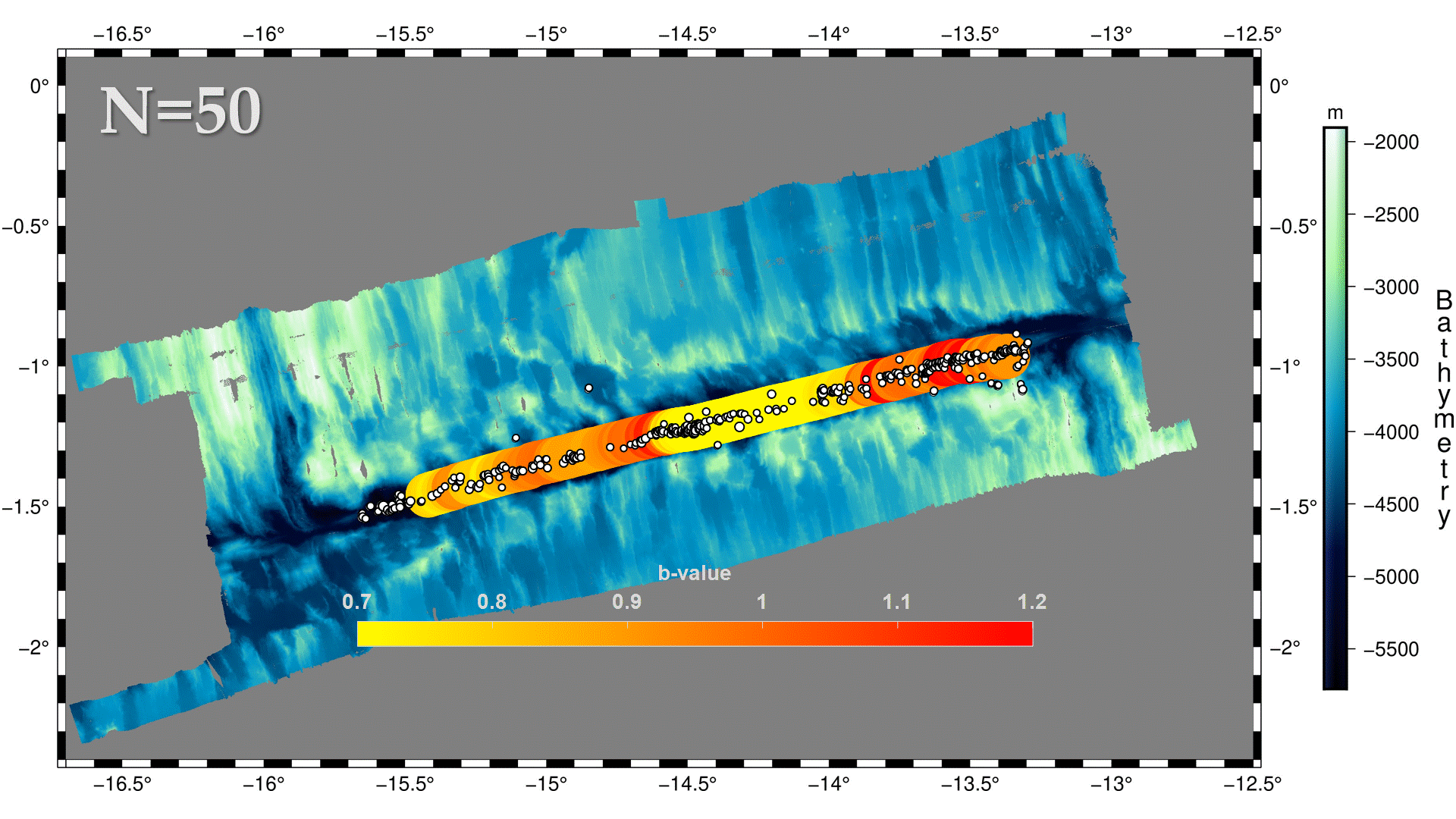

Supplement: Supplementary file 5 — Supplementary Movie 1 [file 41467_2023_41403_MOESM5_ESM.gif]
